# Analyses of the local control of pulmonary Oligometastases after stereotactic body radiotherapy and the impact of local control on survival

**DOI:** 10.1186/s12885-020-07514-9

**Published:** 2020-10-14

**Authors:** Takaya Yamamoto, Yuzuru Niibe, Masahiko Aoki, Takashi Shintani, Kazunari Yamada, Mitsuru Kobayashi, Hideomi Yamashita, Masatoki Ozaki, Yoshihiko Manabe, Hiroshi Onishi, Katsuya Yahara, Atsushi Nishikawa, Kuniaki Katsui, Ryoong-Jin Oh, Atsuro Terahara, Keiichi Jingu

**Affiliations:** 1 Department of Radiation Oncology, Tohoku University Graduate School of Medicine, 1-1 Seiryo-machi, Aoba-ku, Sendai, 980-8574 Japan; 2 Department of Radiology, Toho University Omori Medical Center, 6-11-1 Omorinishi, Ota-ku, Tokyo, 143-8540 Japan; 3 Department of Public Health, Kurume University School of Medicine, 67 Asahi-machi, Kurume, 830-0011 Japan; 4 Department of Radiation Oncology, Hirosaki University, 1 Bunkyo-cho, Hirosaki, 036-8560 Japan; 5 Department of Radiation Oncology and Image-Applied Therapy, Kyoto University Graduate School of Medicine, 54 Shogoinkawahara-cho, Sakyo-ku, Kyoto, 606-8501 Japan; 6 Department of Radiation Oncology, Seirei Mikatahara General Hospital, 3453 Mikatahara-cho, Kita-ku, Hamamatsu, 433-8558 Japan; 7 Department of Radiation Oncology, Fukuyama City Hospital, 5-23-1 Zao-cho, Fukuyama, 721-8511 Japan; 8 Department of Radiology, University of Tokyo, 7-3-1 Hongo, Bunkyo-ku, Tokyo, 113-8655 Japan; 9 Department of Radiation Oncology, Shizuoka City Shimizu Hospital, 1231 Miyakami, Shimizu-ku, Shizuoka, 424-8636 Japan; 10 Department of Radiology, Nagoya City University, 1 Kawasumi, Mizuho-cho, Mizuho-ku, Nagoya, 467-8601 Japan; 11 Department of Radiology, Yamanashi University, 1110 Shimokato, Chuo, 409-3898 Japan; 12 Department of Radiology, University of Occupational and Environmental Health, 1-1 Iseigaoka, Yahatanishi-ku, Kitakyushu, 807-8555 Japan; 13 Department of Radiation Oncology, Shikoku Cancer Center, 160 Minamiumemoto-machi, Matsuyama, 791-0280 Japan; 14 Department of Proton Beam Therapy, Okayama University, 2-5-1 Shikata-cho, Kitaku, Okayama, 700-8558 Japan; 15 Department of Radiology, Miyakojima IGRT Clinic, 1-16-22 Miyakojima-hondori, Miyakojima-ku, Osaka, 534-0021 Japan

**Keywords:** Pulmonary oligometastases, Oligo-recurrence, Sync-oligometastases, Stereotactic body radiotherapy, Local control, Metastasis-directed therapy

## Abstract

**Background:**

Successful local therapy for oligometastases may lead to longer survival. The purpose of this multicentre retrospective study was to investigate factors affecting the local control (LC) of pulmonary oligometastases treated by stereotactic body radiotherapy (SBRT) and to investigate the impact of LC on survival.

**Methods:**

The inclusion criteria included 1 to 5 metastases, the primary lesion and other extrathoracic metastases were controlled before SBRT, and the biological effective dose (BED_10_) of the SBRT was 75 Gy or more. The Cox proportional hazards model was used for analyses.

**Results:**

Data of 1378 patients with 1547 tumours from 68 institutions were analysed. The median follow-up period was 24.2 months. The one-year, 3-year and 5-year LC rates were 92.1, 81.3 and 78.6%, respectively, and the 1-year, 3-year and 5-year overall survival rates were 90.1, 60.3 and 45.5%, respectively. Multivariate analysis for LC showed that increased maximum tumour diameter (*p* = 0.011), type A dose calculation algorithm (*p* = 0.005), shorter overall treatment time of SBRT (*p* = 0.035) and colorectal primary origin (*p* < 0.001 excluding oesophagus origin) were significantly associated with a lower LC rate. In the survival analysis, local failure (*p* < 0.001), worse performance status (1 vs. 0, *p* = 0.013; 2–3 vs. 0, p < 0.001), oesophageal primary origin (vs. colorectal origin, *p* = 0.038), squamous cell carcinoma (vs. adenocarcinoma, *p* = 0.006) and increased maximum tumour diameter (p < 0.001) showed significant relationships with shorter survival.

**Conclusions:**

Several factors of oligometastases and SBRT affected LC. LC of pulmonary oligometastases by SBRT showed a significant survival benefit compared to patients with local failure.

## Background

During the past few decades, increasing attention has been paid to the importance of controlling the primary site in metastatic disease. Some prospective trials and many retrospective studies of metastatic disease have shown improvement in the survival of patients treated with surgery or radiotherapy for the primary lesion, although systemic therapy has been the standard treatment [1–5]. In addition, aside from the activity of the primary lesion, a few metastases known as oligometastases, which might be good candidates for metastasis-directed therapy, have gradually been recognized [6]. The survival benefit of primary lesion control was only revealed in prostate cancer patients with a low metastatic burden (probably an oligometastatic state) [7]. Some phase 2 studies have revealed that intensive local therapy for the primary lesion and for all known oligometastases improved overall survival and disease-free survival of patients, and the results of a future phase 3 trial are awaited [8–10].

In any case, control of the primary lesion is important in the oligometastatic state, and therefore, the classification of oligometastases according to the activity of the primary lesion is important when metastasis-directed therapy is to be performed. Oligometastases have been classified into oligo-recurrences and sync-oligometastases according to the activity of the primary lesion at the time of the initial appearance of oligometastases, and patients with oligo-recurrences have longer survival than those with sync-oligometastases [11, 12]. Recently, an investigation to determine whether there is a survival difference between patients with pulmonary oligo-recurrences and patients with sync-oligometastases after establishing control of the primary lesion was performed in Japan, and it was shown by our group that patients with oligo-recurrences had a survival advantage compared with those with sync-oligometastases [13]. The next question is whether successful local therapy for pulmonary oligometastases leads to longer survival or not and what factors affect local control (LC).

The aim of this study was to identify factors affecting LC and to determine the survival benefit of LC after stereotactic body radiotherapy (SBRT) for pulmonary oligometastases. LC was the secondary endpoint, and this study was a secondary endpoint analysis. In addition, the effect of LC on survival was investigated through an exploratory survival analysis of this large survey.

## Methods

### Eligibility and event definitions

The inclusion criteria were as follows: SBRT for pulmonary oligometastases was performed between January 2004 and June 2015, the number of metastasis was limited to 1 to 5 at the time of the emergence of the SBRT-targeted tumour, the primary lesion and other extrathoracic lesions were controlled before SBRT was performed, and the biological effective dose (BED_10_) of SBRT was 75 Gy or more and the dose per fraction was 4 Gy or more. When there were multiple oligometastases, combination treatment with SBRT for some oligometastases and surgery for other oligometastases was allowed. The exclusion criterion was local recurrence of a primary thoracic tumour. The following formula was used for the calculation of the BED_10_: BED = nd [1 + d/(α/β)], where n is the number of fractions, d is dose per fraction and the α/β ratio is 10 Gy for the tumours. Pulmonary oligometastasis was defined as the appearance of a solid tumour in the lung at the same time as or after treatment of the primary lesion. Local failure was defined as progression of the irradiated tumour which was based on Response Evaluation Criteria in Solid Tumors (RECIST) version 1.1 and further work-up such as ^18^F-Fluorodeoxyglucose Positron Emission Tomography (FDG-PET), biopsy or close follow-up CT scan was done when it was difficult to distinguish progression of the irradiated tumor from lung consolidation. Finally the judgement was done by doctors in charge of the primary disease and radiation oncologist. LC was defined as freedom from local failure, and the locally controlled cohort was defined as freedom from any local failure of the irradiated tumour.

### Ethics approval

This study was a retrospective, multicentre study in Japan. This study was conducted in 68 institutions in Japan. All of the institutions were health insurance-covered medical institutions that cover all citizens in Japan. This study was approved by the ethical committee of a senior facility (Ethics Committee of Toho University Omori Medical Center, reference number: 27–148). The need for informed consent was waived due to the study design, but all participating institutions were guaranteed the chance to opt-out of participating in this study by spreading information about the study via the Internet or posters, and opt-out consent was obtained from all patients.

### Statistical analysis

Statistical analyses were performed using EZR, version 1.37 (Saitama Medical Center, Jichi Medical University, Saitama, Japan), a modified version of R commander (R Foundation for Statistical Computing, Vienna, Austria) [14]. Time to event was calculated from the first day of SBRT to the day an event was confirmed. When no event occurred (local failure or death), the last date of survival (typically the last consultation day) was used as the last moment of follow-up. The cumulative LC and overall survival (OS) rates were calculated using the Kaplan-Meier method, and the 95% confidence interval (95% CI) was calculated using Greenwood’s formula. The Cox proportional hazards model was adjusted for LC analyses, and variables with a *p*-value of < 0.200 in the univariate analyses (UVA) were regarded as potential factors and were entered into the multivariate analysis (MVA) with a stepwise backward elimination/forward addition approach using the Akaike information criterion (AIC) to build the best MVA model. A p-value < 0.050 was defined as significant.

Analyses of pretreatment prognostic factors for OS (the primary endpoint) have been previously reported elsewhere [13]. In this study, LC analyses were performed as secondary endpoint analyses, and the impact of LC on survival was determined by exploratory survival analyses. Because local failure was the observed factor after SBRT, further analysis of OS was performed in this study to determine the effect of LC on OS. First, LC was analysed as a time-dependent covariate. Previously reported covariates selected by the stepwise approach without changing the continuous variables into categorical variables were used: performance status (PS), primary lesions, pathology of the primary lesion, oligometastatic state and maximum tumour diameter. Local status was forced into this multivariate model as a time-dependent covariate. Second, for the sensitivity analyses, the landmark analysis method was used [15, 16]. There was thought to be a time-to-failure bias that patients with local failure must survive at least until the time of the confirmation of local failure; in other words, the separation of the locally controlled and locally failed cohorts was affected by the follow-up period. Therefore, the landmark method was used: 6 months, 1 year, 2 years and 3 years were set as the landmark times, and all local failures after the landmark time and all deaths before that time were ignored. Then, a log-rank test was applied to compare locally controlled and locally failed cohorts with a *p*-value of ≤0.012 denoting significance. Lung adverse events (AEs) were graded using the National Cancer Institute Common Terminology Criteria for Adverse Events version 4.0.

## Results

### Patient characteristics

A total of 1378 patients with 1547 tumours from 68 institutions were enrolled in this study. The baseline characteristics of the patients, primary tumours, oligometastatic tumours and SBRT are summarized in Table 1. PS and additional chemotherapy were judged at the timing of each SBRT if SBRT was performed asynchronously for two or more tumours. The dose calculation algorithm of type B was equivalent to the Analytical Anisotropic Algorithm, type C was equivalent to the Monte Carlo Algorithm and type A was an older generation algorithm, such as the Pencil Beam Convolution. More than half of oligometastatic tumours were treated with 4-fraction SBRT (959 tumours), and the median overall treatment time (OTT) of 4-fraction SBRT was 5 days. The most typical prescription dose was 48 Gy in 4-fraction to the isocenter (372 tumours).

**Table 1:** Characteristics of patients, primary tumours, oligometastatic tumours and SBRT characteristics

Characteristic	Distribution	Number (%)
All tumours		1547
Sex	Male	994 (64.3)
	Female	553 (35.7)
Age, years	Median, range, IQR	72; 16–93; 63–78
ECOG Performance Status	0	841 (54.4)
	1	529 (34.2)
	2	90 (5.8)
	3	19 (1.2)
	Missing	68 (4.4)
Primary lesion sites	Lung	451 (29.2)
	Colorectum	391 (25.3)
	Head and Neck	126 (8.1)
	Oesophagus	132 (8.5)
	Others	447 (28.9)
Pathology of primary lesion	Adenocarcinoma	861 (55.7)
	Squamous cell carcinoma	396 (25.6)
	Sarcoma	47 (3.0)
	Others	168 (10.9)
	Missing	75 (4.8)
Control method of primary lesion	Surgery	1222 (79.0)
	Chemoradiation	130 (8.4)
	Radiation (X-ray or particle)	70 (4.5)
	Others	40 (2.6)
	Missing	85 (5.5)
Disease-free interval, months	Median, range, IQR	17.5; 0–423.9; 8.0–34.3
Oligometastatic state	Oligo-recurrences	1157 (74.8)
	Sync-oligometastases	133 (8.6)
	Unclassified	133 (8.6)
	Missing	124 (8.0)
SBRT performed institution	Academic	642 (41.5)
	Non-academic	905 (58.5)
Date SBRT was performed	2004–2009	518 (33.5)
	2010–2015	1029 (66.5)
Chemotherapy	Before SBRT	Yes, 591 (38.2) No, 945 (61.1) Missing, 11 (0.7)
	Concurrent with SBRT	Yes, 34 (2.2) No, 1513 (97.8)
	After SBRT	Yes, 242 (15.6) No, 998 (64.5) Missing, 307 (19.9)
Maximum tumour diameter, cm	Median, range, IQR	1.5; 0.3–6.5; 1.0–2.0
Number of oligometastases at the time of emergence of the SBRT-targeted tumour	1	1036 (67.0)
	2–5	503 (32.5)
	Missing	8 (0.5)
Lung lobe involved with treated tumour	Right upper lobe	293 (18.9)
	Right middle lobe	83 (5.4)
	Right lower lobe	321 (20.8)
	Left upper lobe	294 (19.0)
	Left lower lobe	226 (14.6)
	Unknown lobe	Right lung, 12; Left lung, 7
	Missing	311 (20.1)
Beams	Multiple static	1145 (74.0)
	Arc	401 (25.9)
	Missing	1 (0.1)
X-ray energy	4 MV only	202 (13.1)
	6 MV only	1179 (76.2)
	Others	160 (10.3)
	Missing	6 (0.4)
Field coplanarity	Coplanar field	1139 (73.6)
	Non-coplanar field	404 (26.1)
	Missing	4 (0.3)
Dose calculation algorithm	Type A	541 (35.0)
	Type B	799 (51.6)
	Type C	144 (9.3)
	Missing	63 (4.1)
Dose prescription	IC	1103 (71.3)
	D95 of PTV	317 (20.5)
	Others	127 (8.2)
BED_10_ at isocenter, Gy	Median, range, IQR	105.6; 75.0–289.5; 105.6–126.9
Number of SBRT fractions	2–3	27 (1.7)
	4	959 (62.0)
	5	236 (15.3)
	6–16	324 (20.9)
	missing	1 (0.1)
OTT of SBRT, day	Median, range, IQR	7; 3–81; 4–11

Abbreviations: *SBRT* Stereotactic body radiotherapy, *IQR* Interquartile range, *ECOG* Eastern Cooperative Oncology Group, *IC* Isocenter, *D95 of PTV* Dose covering 95% of planning target volume, *BED* Biological effective dose, *OTT* Overall treatment time

### Treatment outcomes

**Fig. 1:**
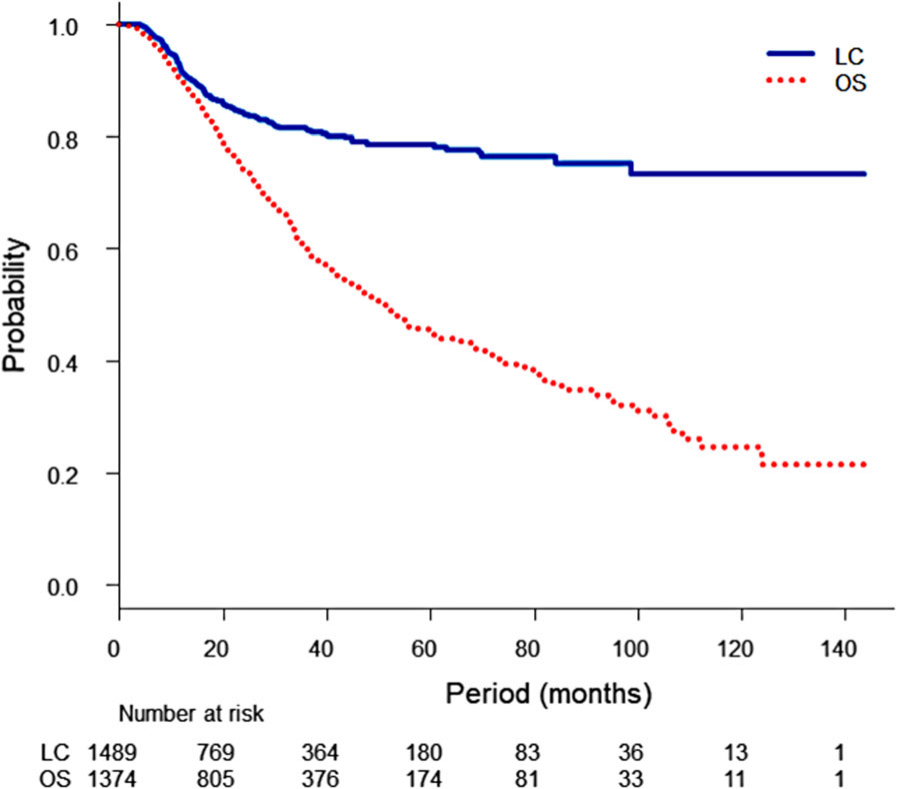
Kaplan-Meier local control (LC) curve and overall survival (OS) curve. The results of the Kaplan-Meier LC curves and OS curves are shown. Because of the missing values, 1374 patients with 1489 tumours were used for the estimate

The median follow-up period for all patients was 24.2 months (range, 0.1–143.6 months; interquartile range [IQR], 13.7–42.7 months), and that for survivors was 26.9 months (range, 0.1–143.6 months; IQR, 14.7–49.4 months). The estimated 1-year, 3-year and 5-year LC rates were 92.1% (95% CI, 90.4–93.4%), 81.3% (95% CI, 78.8–83.6%) and 78.6% (95% CI, 75.6–81.2%), respectively (Fig. 1). Local failure of the irradiated tumour occurred in 222 tumours, and the median interval from SBRT to local failure was 12.4 months (range, 2.9–98.6 months; IQR, 9.1–19.7 months). A total of 536 patients with 603 tumours died, and 10 deaths were caused by AEs of SBRT. The estimated 1-year, 3-year and 5-year OS rates were 90.1% (95% CI, 88.3–91.6%), 60.3% (95% CI, 57.1–63.3%) and 45.5% (95% CI, 41.8–49.1%), respectively (Fig. 1). The median survival period was 51.4 months (95% CI, 45.0–55.7 months). There were lung AEs reports from 1200 tumours in 1040 patients. Of those patients, 122 patients with 143 tumours (11.7%) had grade 2 or higher and 26 patients with 32 tumours (2.5%) had grade 3 or higher. Among 10 patients who developed AEs of grade 5, 3 patients with 4 tumours had grade 5 haemoptysis, and grade 5 radiation pneumonitis occurred in 7 patients with 9 tumours who had the following features: 3 patients had coexisting interstitial pneumonia, 3 patients had received thoracic radiotherapy before or after SBRT and one patient had a solitary metastasis but an older age (87 years).

### Analyses for LC and survival

**Fig. 2:**
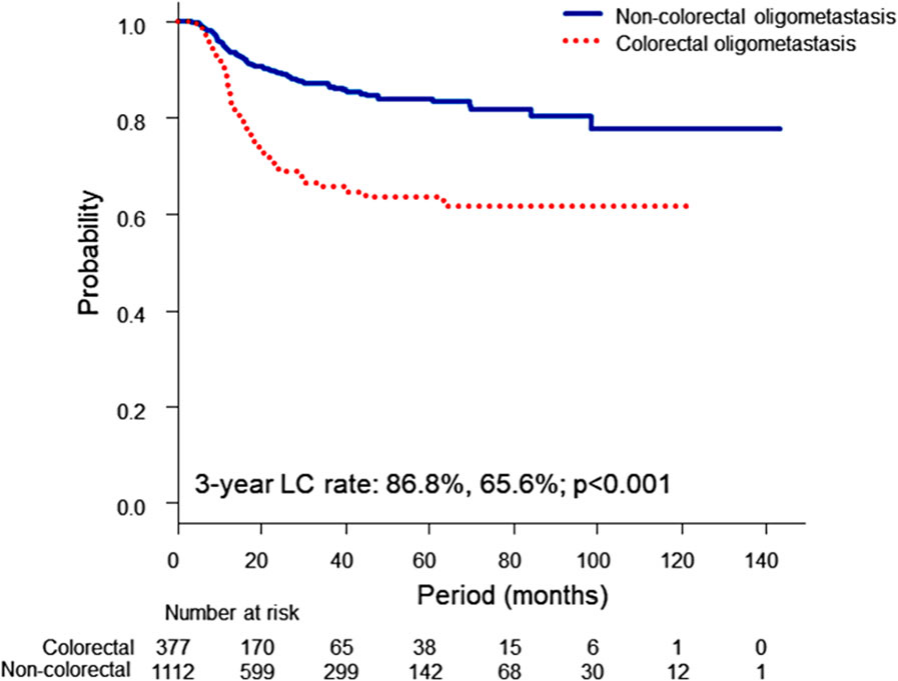
Kaplan-Meier local control curves according to the primary lesion: colorectal oligometastasis and other (non-colorectal) oligometastasis. Local control of pulmonary oligometastases from the colorectum was far worse than that of pulmonary oligometastases of non-colorectal origin

The UVA results for LC are shown in Supplementary Table 1, and the MVA results for LC are shown in Table 2. Maximum tumour diameter (per 1-cm increase; hazard ratio [HR], 1.297; 95% CI, 1.059–1.588; *p* = 0.011), type B dose calculation algorithm (ref. type A; HR, 0.592; 95% CI, 0.410–0.856; *p* = 0.005), OTT of SBRT (per 10-day prolongation; HR, 0.610; 95% CI, 0.385–0.966; *p* = 0.035) and primary lesions emerged as significant factors, showing that a higher HR was related to a higher rate of local failure. In regard to primary lesions, the LC rate for oligometastases from colorectal cancer was significantly lower than the LC rate for oligometastases from lung cancer (ref. colorectum; HR, 0.413; 95% CI, 0.274–0.622; *p* < 0.001), head and neck cancer (ref. colorectum; HR, 0.194; 95% CI, 0.077–0.489; *p* < 0.001) and other cancers (ref. colorectum; HR, 0.337; 95% CI, 0.208–0.546; p < 0.001), excluding oesophagus (ref. colorectum; HR, 0.618; 95% CI, 0.306–1.248; *p* = 0.179). Kaplan-Meier LC curves according to oligometastases from colorectal cancer and oligometastases from other cancers are shown in Fig. 2 (*p* < 0.001).

**Table 2:** Multivariate Cox regression analysis of local control and overall survival

Factors	Covariate	Local control		Overall survival	
		HR (95% CI)	*P* value	HR (95% CI)	P value
Local status	Controlled	–	–	reference	
	Failed	–	–	2.390 (1.839–3.106)	< 0.001
ECOG PS	0	–	–	reference	
	1	–	–	1.316 (1.059–1.635)	0.013
	2–3	–	–	2.008 (1.405–2.869)	< 0.001
Primary lesion sites	Colorectum	reference		reference	
	Lung	0.413 (0.274–0.622)	< 0.001	0.936 (0.685–1.280)	0.681
	H&N	0.194 (0.077–0.489)	< 0.001	0.905 (0.556–1.474)	0.690
	Oesophagus	0.618 (0.306–1.248)	0.179	1.650 (1.027–2.650)	0.038
	Others	0.337 (0.208–0.546)	< 0.001	1.208 (0.863–1.691)	0.270
Pathology of primary lesion	Adenoca.	–	–	reference	
	SqCC	–	–	1.525 (1.122–2.072)	0.006
	Others	–	–	1.291 (0.916–1.818)	0.143
Oligometastatic state	Oligo-rec	–	–	reference	
	Sync-oligo	–	–	1.391 (0.988–1.960)	0.058
	Unclassified	–	–	1.246 (0.905–1.714)	0.177
Chemotherapy concurrent with SBRT	Yes	1.969 (0.859–4.513)	0.109	–	–
	No	reference		–	–
Maximum tumour diameter	Per 1 cm	1.297 (1.059–1.588)	0.011	1.266 (1.131–1.417)	< 0.001
Dose calculation algorithm	Type A	reference		–	–
	Type B	0.592 (0.410–0.856)	0.005	–	–
	Type C	0.732 (0.371–1.445)	0.368	–	–
BED_10_ at isocenter	Per 10 Gy	0.912 (0.828–1.006)	0.065	–	–
OTT of SBRT	Per 10 days	0.610 (0.385–0.966)	0.035	–	–

Abbreviations: *HR* Hazard ratio, *CI* Confidence interval, *ECOG* Eastern Cooperative Oncology Group, *PS* Performance status, *H&N* Head and neck, *Adenoca.* Adenocarcinoma, *SqCC* Squamous cell carcinoma, *Oligo-rec.* Oligo-recurrences, *Sync-oligo* Sync-oligometastases, *SBRT* Stereotactic body radiotherapy, *BED* Biological effective dose, *OTT* Overall treatment time

**Fig. 3:**
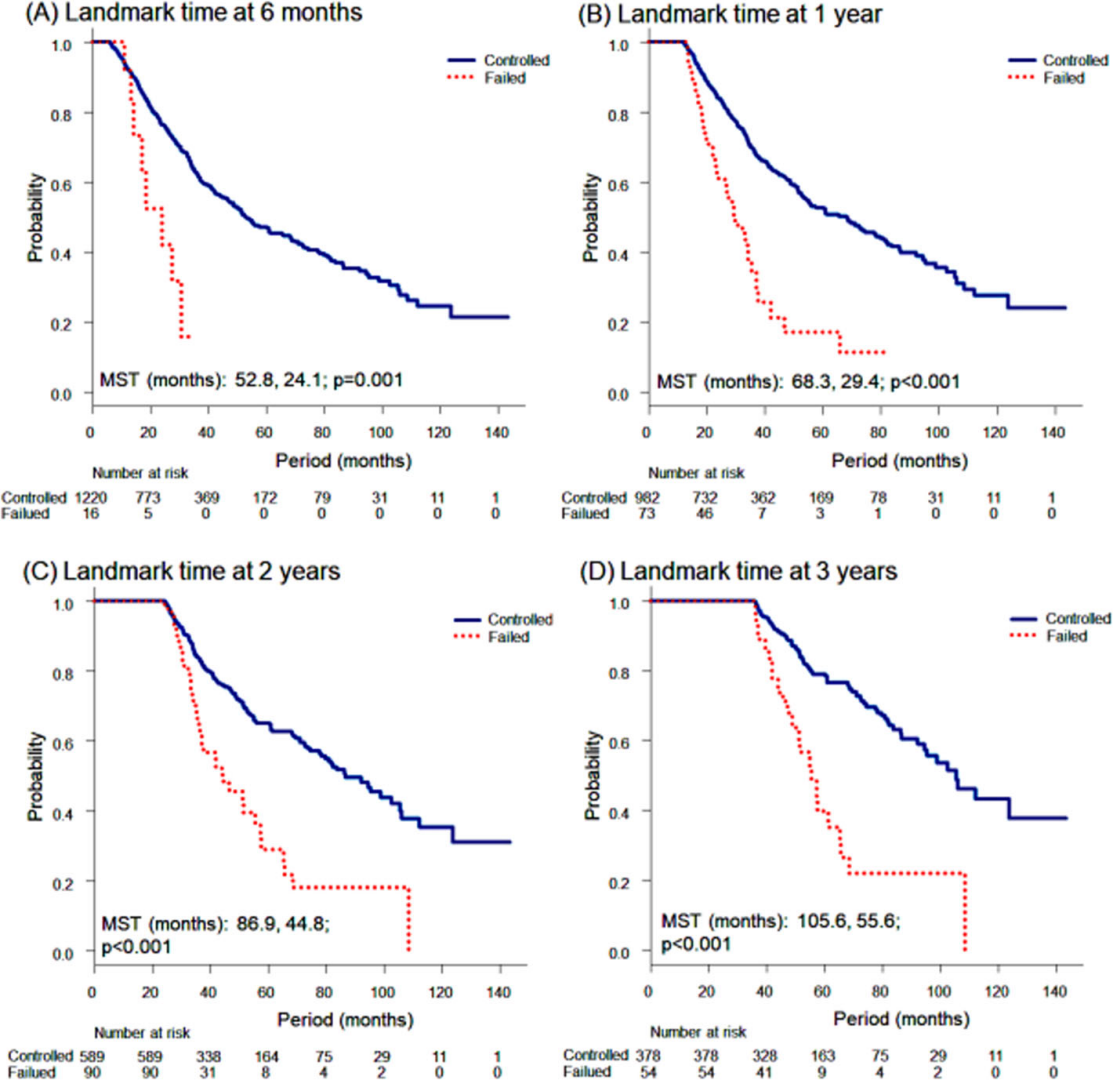
Landmark analyses for OS to compare between a locally controlled cohort and a locally failed cohort. All the local failures after the landmark time and all the deaths before that time were ignored. The locally controlled group showed a consistent significantly longer survival than the locally failed group at each landmark time

In the OS analyses, there were significant relationships of a poor survival rate with local failed cohort (ref. local controlled cohort; HR, 2.390; 95% CI, 1.839–3.106; *p* < 0.001), PS of 1 (ref. PS of 0; HR, 1.316; 95% CI, 1.059–1.635; *p* = 0.013), PS of 2–3 (ref. PS 0; HR, 2.008; 95% CI, 1.405–2.869; p < 0.001), oligometastases from the oesophagus (ref. colorectum; HR, 1.650; 95% CI, 1.027–2.650; *p* = 0.038), squamous cell carcinoma pathology of the primary lesion (ref. adenocarcinoma; HR, 1.525; 95% CI, 1.122–2.072; *p* = 0.006) and maximum oligometastatic tumour diameter (per 1-cm increase; HR, 1.266; 95% CI, 1.131–1.417; p < 0.001; Table 2). On the other hand, sync-oligometastases showed marginal significance (ref. oligo-recurrence, HR, 1.391; 95% CI, 0.988–1.960; *p* = 0.058). In the landmark analyses, the LC status of the SBRT sites showed significant differences between the local controlled group and the local failed group at all landmark time points (*p* ≤ 0.001 at each point). The locally controlled group showed consistently longer survival than the locally failed group (Fig. 3).

## Discussion

This study revealed the independent significance of the OS benefit in a locally controlled cohort compared to that in a locally failed cohort by using SBRT for pulmonary oligometastases. In colorectal cancer, local failure of irradiated metastases has been reported to have a correlation with worse OS [17]. Analyses of the large survey data in this study expanded the evidence to oligometastases from various primary cancer types. It is certain that there is a situation in which metastasis-directed therapy works well and contributes to longer survival; therefore, LC of metastatic lesions is important in an oligometastatic situation. The LC rate for patients who received SBRT for pulmonary metastases has been shown to be relatively high in prospective trials, but similar results have not always been obtained in a real-world setting [18–20]. The LC analyses performed in this study provide several key factors for successful LC by SBRT.

It needs to be emphasized that SBRT should be given to appropriate patients. In the present study, among the inclusion criteria was that the primary lesion and extrathoracic lesions needed to be controlled before SBRT and all pulmonary oligometastases needed to be treated with local therapy. In a retrospective study in patients with synchronous oligometastatic (probably sync-oligometastases) epidermal growth factor receptor (EGFR)-mutant non-small cell lung cancer who were treated with an EGFR-tyrosine kinase inhibitor, OS improved only in patients who received local ablative therapy for the primary lesion and for all oligometastatic lesions [21]. Thus, the number of metastases and the treatability of all lesions by local therapy are important. In metastatic prostate cancer, a survival benefit from definitive radiotherapy for the primary lesion was observed only among those with a low metastatic burden, and additional radiotherapy for all oligometastases showed retrospectively better castration-resistant prostate cancer-free survival than radiotherapy for only the primary lesion [7, 22]. A recent consensus report proposed a maximum of five metastases and three organs as synchronous oligometastatic non-small cell lung cancer (probably sync-oligometastases) [23]. To provide metastasis-directed therapy well, recent phase 2 trials required thoughtful eligibility criteria, treating the primary lesion and all known oligometastases with local therapy [8–10, 24]. Appropriate selection of patients is important to obtain benefit from SBRT.

The collaborative and detailed analyses of this study have also revealed some factors that affect LC. The MVA for LC showed that the primary site, maximum tumour diameter treated by SBRT and dose calculation algorithm were significant factors affecting LC, and these factors confirmed previous findings [25–27]. Poor LC of metastatic lung tumours from the colorectum has been discussed because there are some reports of extremely low LC rates of colorectal metastases as well as reports of low LC rates of liver or bone metastases from the colorectum [27–32]. Interestingly, a German group reported that there was no significant difference in LC rates for colorectal metastases and non-colorectal metastases in the lung, but there was a significant difference in LC rates for those in the liver [20, 33]. In this study, a large crude difference of approximately 20% was found between the LC rates for colorectal oligometastases and non-colorectal oligometastases (Fig. 2). Analysis of SBRT for pulmonary oligometastases from colorectal cancer showed that dose escalation improved LC [34]. Consideration should be given to possible ways to improve LC of colorectal oligometastases.

The OTT of SBRT also showed a significant relationship with LC. The OTT of SBRT probably reflected both intervals of each treatment session of SBRT and the number of SBRT fractions. Considering that the number of fractions was not a significant factor in UVA, the significance of a longer OTT is mainly due to the intervals between each treatment session. A better effectiveness of longer intervals between each treatment session would be reasonable because it might reflect the effect of reoxygenation, which has an influence on tumour radiosensitivity [35, 36]. SBRT sessions are often performed on consecutive days in Japan. However, the Radiation Therapy Oncology Group (RTOG) trial and another study required longer intervals in SBRT [23, 37]. Unfortunately, whether SBRT was performed on consecutive days or non-consecutive days was not investigated in this study, but appropriate intervals between sessions, such as 40 h, might contribute to higher LC rates.

There are several limitations of this study. The retrospective nature of this study made all of the analyses subject to selection bias and confounding by indication. There was a considerable amount of missing data, the SBRT methods varied from centre to centre, the follow-up examinations and the evaluation of local failure were inconsistent, and there were unmeasured or uncontrolled factors. We could not investigate unlisted survey items, such as a central lung tumour or not, or the details of the history of extrathoracic lesions, such as the number of involved organs, patient comorbidities and dose-volume histogram analyses of the lung.

## Conclusions

In conclusion, to achieve higher LC of pulmonary oligometastases by SBRT, the use of a type A algorithm should be avoided and a longer OTT of SBRT contributes to a higher LC rate. Tumour characteristics such as small-size oligometastases and non-colorectal oligometastases also showed a higher LC rate. LC of pulmonary oligometastases by SBRT with a controlled primary lesion before SBRT had a survival benefit compared to the locally uncontrolled group, and LC status showed the highest HR in multivariate analysis for OS.

## Supplementary information

**Additional file 1.**

## Data Availability

The dataset used this study are currently unavailable because it contains materials in unpublished manuscripts.

## Abbreviations

LC: Local control
SBRT: Stereotactic body radiotherapy
BED: Biological effective dose
RECIST: Response Evaluation Criteria in Solid Tumors
FDG-PET: ^18^F-Fluorodeoxyglucose Positron Emission Tomography
OS: Overall survival
CI: Confidence interval
UVA: Univariate analysis
MVA: Multivariate analysis
AIC: Akaike information criterion
AE: Adverse event
PS: Performance status
OTT: Overall treatment time
IQR: Interquartile range
HR: Hazard ratio
EGFR: Epidermal growth factor receptor
RTOG: Radiation Therapy Oncology Group
