# Supplementary material for: Analyses of the local control of pulmonary Oligometastases after stereotactic body radiotherapy and the impact of local control on survival
Source: BMC Cancer. 2020 Oct 14;20:997. doi: 10.1186/s12885-020-07514-9 (PMC7559191; doi:10.1186/s12885-020-07514-9)
Supplement: Supplementary file 1 — Additional file 1. [file 12885_2020_7514_MOESM1_ESM.docx]

Supplementary 1. Univariate Cox regression analyses for local control

| Variable | Reference or Unit | HR (95% CI) | p-value |
| --- | --- | --- | --- |
| Age | Per 1-year increase | 0.999 (0.987, 1.011) | 0.910 |
| Sex |  |  |  |
| Male | Female | 1.118 (0.847, 1.475) | 0.431 |
| ECOG PS |  |  |  |
| 1 | 0 | 1.255 (0.950, 1.659) | 0.109 |
| 2-3 | 0 | 0.961 (0.517, 1.784) | 0.899 |
| Primary lesion |  |  |  |
| Lung | Colorectum | 0.436 (0.315, 0.603) | <0.001 |
| Head and Neck | Colorectum | 0.300 (0.161, 0.561) | <0.001 |
| Oesophagus | Colorectum | 0.521 (0.302, 0.896) | 0.018 |
| Others | Colorectum | 0.293 (0.200, 0.429) | <0.001 |
| Pathology of primary lesion |  |  |  |
| Squamous cell carcinoma | Adenocarcinoma | 0.709 (0.507, 0.993) | 0.045 |
| Sarcoma | Adenocarcinoma | 0.230 (0.057, 0.931) | 0.039 |
| Others | Adenocarcinoma | 0.523 (0.308, 0.890) | 0.016 |
| Disease-free interval | Per 10-month increase | 0.958 (0.908, 1.011) | 0.118 |
| Oligometastatic state |  |  |  |
| Sync-oligometastasis | Oligo-recurrence | 0.837 (0.485, 1.445) | 0.524 |
| Unclassified | Oligo-recurrence | 1.125 (0.707-1.789) | 0.618 |
| Control method of primary lesion |  |  |  |
| Surgery | Others | 2.468 (0.612, 9.943) | 0.204 |
| (Chemo)Radiation | Others | 1.958 (0.460, 8.329) | 0.363 |
| SBRT treatment date |  |  |  |
| 2010-2015 | 2004-2009 | 0.729 (0.556, 0.955) | 0.022 |
| Chemotherapy |  |  |  |
| Before SBRT | Not administered | 0.974 (0.740, 1.283) | 0.852 |
| Concurrent with SBRT | Not administered | 1.898 (0.973, 3.699) | 0.059 |
| After SBRT | Not administered | 1.016 (0.697, 1.479) | 0.934 |
| Maximum tumour diameter | Per 1-cm increase | 1.317 (1.141, 1.521) | <0.001 |
| Number of oligometastases |  |  |  |
| 1 | 2-5 | 1.447 (1.066, 1.965) | 0.017 |
| Irradiated tumour location |  |  |  |
| Lower lobe | Upper or middle lobe | 0.900 (0.664, 1.221) | 0.500 |
| Beams |  |  |  |
| Multiple static | Arc | 0.846 (0.631, 1.134) | 0.263 |
| X-ray energy |  |  |  |
| 6 MV only | 4 MV only | 0.976 (0.661, 1.441) | 0.904 |
| Others | 4 MV only | 0.859 (0.478, 1.541) | 0.610 |
| Field coplanarity |  |  |  |
| Non-coplanar field | Coplanar field | 1.144 (0.838, 1.562) | 0.395 |
| Dose calculation algorithm |  |  |  |
| Type B | Type A | 0.699 (0.527, 0.926) | 0.012 |
| Type C | Type A | 0.635 (0.368, 1.094) | 0.101 |
| Dose prescription |  |  |  |
| Isocenter | D95 of PTV | 1.802 (1.209, 2.685) | 0.003 |
| Others | D95 of PTV | 1.255 (0.670, 2.349) | 0.478 |
| BED_10_ at isocenter | Per 10-Gy increase | 0.942 (0.878, 1.010) | 0.095 |
| Fraction | Per 1-fraction | 0.969 (0.904, 1.040) | 0.387 |
| Overall treatment time of SBRT | Per 10-day prolongation | 0.774 (0.524-1.144) | 0.199 |

Abbreviations: HR, hazard ratio; CI, confidence interval; ECOG, Eastern Cooperative Oncology Group; PS, performance status; SBRT, stereotactic body radiotherapy; D95 of PTV, dose covering 95% of planning target volume; BED, biological effective dose.
